# Viscoelastic properties of colorectal liver metastases reflect tumour cell viability

**DOI:** 10.1186/s12967-024-05559-z

**Published:** 2024-08-16

**Authors:** Lisa-Marie Skrip, Simon Moosburner, Peter Tang, Jing Guo, Steffen Görner, Heiko Tzschätzsch, Kristin Brüggemann, Kilian Alexander Walter, Clarissa Hosse, Uli Fehrenbach, Alexander Arnold, Dominik Modest, Felix Krenzien, Wenzel Schöning, Thomas Malinka, Johann Pratschke, Björn Papke, Josef A. Käs, Ingolf Sack, Igor M. Sauer, Karl H. Hillebrandt

**Affiliations:** 1 Department of Surgery, Charité – Universitätsmedizin Berlin, Corporate Member of Freie Universität Berlin and Humboldt- Universität zu Berlin, Augustenburger Platz 1, 13353 Berlin, Germany; 2 Clinician Scientist Program, Berlin Institute of Health at Charité – Universitätsmedizin Berlin, BIH Academy, Berlin, Germany; 3 Department of Radiology, Charité – Universitätsmedizin Berlin, Corporate Member of Freie Universität Berlin and Humboldt- Universität zu Berlin, Charitéplatz 1, 10117 Berlin, Germany; 4 Institute of Medical Informatics, Charité – Universitätsmedizin Berlin, Corporate Member of Freie Universität Berlin and Humboldt- Universität zu Berlin, Charitéplatz 1, 10117 Berlin, Germany; 5 Institute of Pathology, Charité – Universitätsmedizin Berlin, Corporate Member of Freie Universität Berlin and Humboldt- Universität zu Berlin, Charitéplatz 1, 10117 Berlin, Germany; 6 Department of Hematology, Oncology, and Cancer Immunology (CVK/CCM), Charité – Universitätsmedizin Berlin, Corporate Member of Freie Universität Berlin and Humboldt- Universität zu Berlin, Charitéplatz 1, 10117 Berlin, Germany; 7 Soft Matter Physics Division, Peter Debye Institute for Soft Matter Physics, University of Leipzig, Leipzig, Germany

**Keywords:** Magnetic resonance elastography, Colorectal liver metastases, Preoperative chemotherapy, Regression

## Abstract

**Background:**

Colorectal cancer is the third most common tumour entity in the world and up to 50% of the patients develop liver metastases (CRLM) within five years. To improve and personalize therapeutic strategies, new diagnostic tools are urgently needed. For instance, biomechanical tumour properties measured by magnetic resonance elastography (MRE) could be implemented as such a diagnostic tool. We postulate that ex vivo MRE combined with histological and radiological evaluation of CRLM could provide biomechanics-based diagnostic markers for cell viability in tumours.

**Methods:**

34 CRLM specimens from patients who had undergone hepatic resection were studied using ex vivo MRE in a frequency range from 500 Hz to 5300 Hz with increments of 400 Hz. Single frequency evaluation of shear wave speed and wave penetration rate as proxies for stiffness and viscosity was performed, along with rheological model fitting based on the spring-pot model and powerlaw exponent α, ranging between 0 (complete solid behaviour) and 1 (complete fluid behaviour). For histological analysis, samples were stained with H&E and categorized according to the degree of regression. Quantitative histologic analysis was performed to analyse nucleus size, aspect ratio, and density. Radiological response was assessed according to RECIST-criteria.

**Results:**

Five samples showed major response to chemotherapy, six samples partial response and 23 samples no response. For higher frequencies (> 2100 Hz), shear wave speed correlated significantly with the degree of regression (*p* ≤ 0.05) indicating stiffer properties with less viable tumour cells. Correspondingly, rheological analysis of *α* revealed more elastic-solid tissue properties at low cell viability and major response (α = 0.43 IQR 0.36, 0.47) than at higher cell viability and no response (α = 0.51 IQR 0.48, 0.55; *p* = 0.03). Quantitative histological analysis showed a decreased nuclear area and density as well as a higher nuclear aspect ratio in patients with major response to treatment compared to patients with no response (all *p* < 0.05).

**Discussion:**

Our results suggest that MRE could be useful in the characterization of biomechanical property changes associated with cell viability in CRLM. In the future, MRE could be applied in clinical diagnosis to support individually tailored therapy plans for patients with CRLM.

## Background

Colorectal cancer (CRC) ranks third in global cancer incidence among men and women [1]. Moreover, it represents the third most frequent cause of cancer-related mortality in men and the fourth in women [1]. The specific localization of the primary tumour has a major impact on the patient’s prognosis. Left-sided colorectal cancer has a higher incidence compared to right-sided and an overall superior outcome [2]. However, an increase in the incidence of right-sided colon cancer has been observed over the last decades, coinciding with a worse overall survival, when adjusted to the tumour stage [2]. The liver represents the most common localisation for metastases [3]. At initial diagnosis, almost one-quarter of the patients already have colorectal liver metastases (CRLM) and up to 50% of the patients will develop metastases within five years, despite being more common in left-sided colorectal cancer [3, 4]. During the 1990s, the two-year overall survival rate was observed to be as low as 21% [5]. Significant advancements in systemic chemotherapy, targeted therapies, and surgical therapeutic options have led to a notable improvement in the survival rates of patients with CRLM [6]. As a result, the 5-year overall patient survival has increased to 35–40% in the last two decades. Nevertheless, there is still an urgent need for novel approaches in order to further reduce the CRLM-related mortality [6]. Among many aspects, accurate and high-quality imaging is essential to monitor the course of the disease to develop personalized therapeutic strategies for patients with CRLM.

The current state-of-the-art imaging modality for the detection and response evaluation of systemic chemotherapy in CRLM is magnetic resonance imaging (MRI) [7]. The importance of MRI in the preoperative setting was underlined by the recently published CAMINO-Trial showing that the treatment plan had to be modified in 31% of the patients after MRI examination [8].

However, a recent study has also shown that approximately 44% of the patients with CRLM show a discrepancy between radiological and pathological response [9]. The combination of radiological response and pathological non-response was associated with a significantly reduced disease-free survival compared to patients with pathological response and a radiological non-response (8.6 months vs. 13.9 months), showing that the currently used imaging modalities might be less accurate than the pathological evaluation to predict the treatment response, which is directly linked to the patient outcome [9]. Although there has been progress in the field of MRI by applying the apparent diffusion coefficient and diffusion weighted imaging parameters to predict pathological response, further diagnostic tools may improve the prediction accuracy [10–12].

One improvement in clinical diagnostics could be achieved by the implementation of magnetic resonance elastography (MRE). MRE is an emerging imaging technique that combines MRI with mechanical vibrations to generate an imaging contrast of viscoelastic tissue properties. This imaging modality can be used to detect and quantify changes in the biomechanical properties of soft tissues due to disease- and therapeutic-induced structural alterations [13]. In vivo MRE typically operates within a vibrations range of 20–80 Hz, thereby encompassing only a limited number of frequencies, while ex vivo MRE is capable to cover a wider frequency range from 500 Hz to 6000 Hz in tissue samples of only a few millimetres of diameter [13, 14].

MRE has been implemented in clinical practice for the assessment of liver fibrosis and cirrhosis, both conditions characterized by a marked increase in liver stiffness [13, 15]. Moreover, other liver pathologies, including various tumours, can induce tissue stiffening. In our recently published study utilizing in vivo MRE, we demonstrated that malignant tumours exhibit elevated stiffness and higher viscosity compared to benign tumours [16]. However, until now MRE has seen limited application in evaluating the treatment response to systemic chemotherapy through the measurement of changes in the tumour viscoelastic properties. To the best of our knowledge, only *Vogl et al.* have shown via in vivo MRE that the stiffness of CRLM increases with time after transarterial chemoembolization [17]. These results indicate that the response to therapy influences the viscoelasticity of CRLM. However, an evaluation of radiological or pathological response was not performed.

Therefore, we conducted an experimental ex vivo study to characterize the viscoelastic properties of colorectal liver metastases in fresh tissue specimen utilizing a broadband 0.5 Tesla tabletop MRE, which was employed in our previous studies [18–20].

Our main hypothesis was that the combination of multifrequency MRE with histological assessment of CRLM could yield insights enabling the utilization of tissue mechanical properties as a diagnostic marker for treatment response.

## Methods

### Sample acquisition and preparation

Tissue specimens were obtained directly from the operation room from patients with CRLM, especially adenocarcinomas, undergoing liver resection at the Department of Surgery, Charité – Universitätsmedizin Berlin, Campus Charité Mitte and Campus Virchow Klinikum. The resected tissue specimens were directly transferred for pathological assessment. A board-certified pathologist dissected the CRLM tissue from the resectate and excised a tissue sample for MRE and histological analysis as subsequent investigations, if the sample size was sufficient. The CRLM samples were wrapped within moist compresses, dampened with phosphate-buffered saline, and stored at 4 °C until further use to avoid drying up and keep the viscoelastic properties as unaltered as possible, as it has been established in previous studies of our research group [21, 22].

The CRLM samples were collected between May 2022 und May 2023. Out of 46 measured samples using MRE, 34 could be evaluated successfully. A major problem was the necessary diameter of the tissue for the MRE measurements. This prospective study was approved by the ethical committee of the Charité (approvals: EA1/214/19 and EA4/132/22). The written informed consent was obtained from all patients included in the study. The study was conducted according to the ethical standards of the Helsinki Declaration.

### MRE measurements

**Fig. 1:**
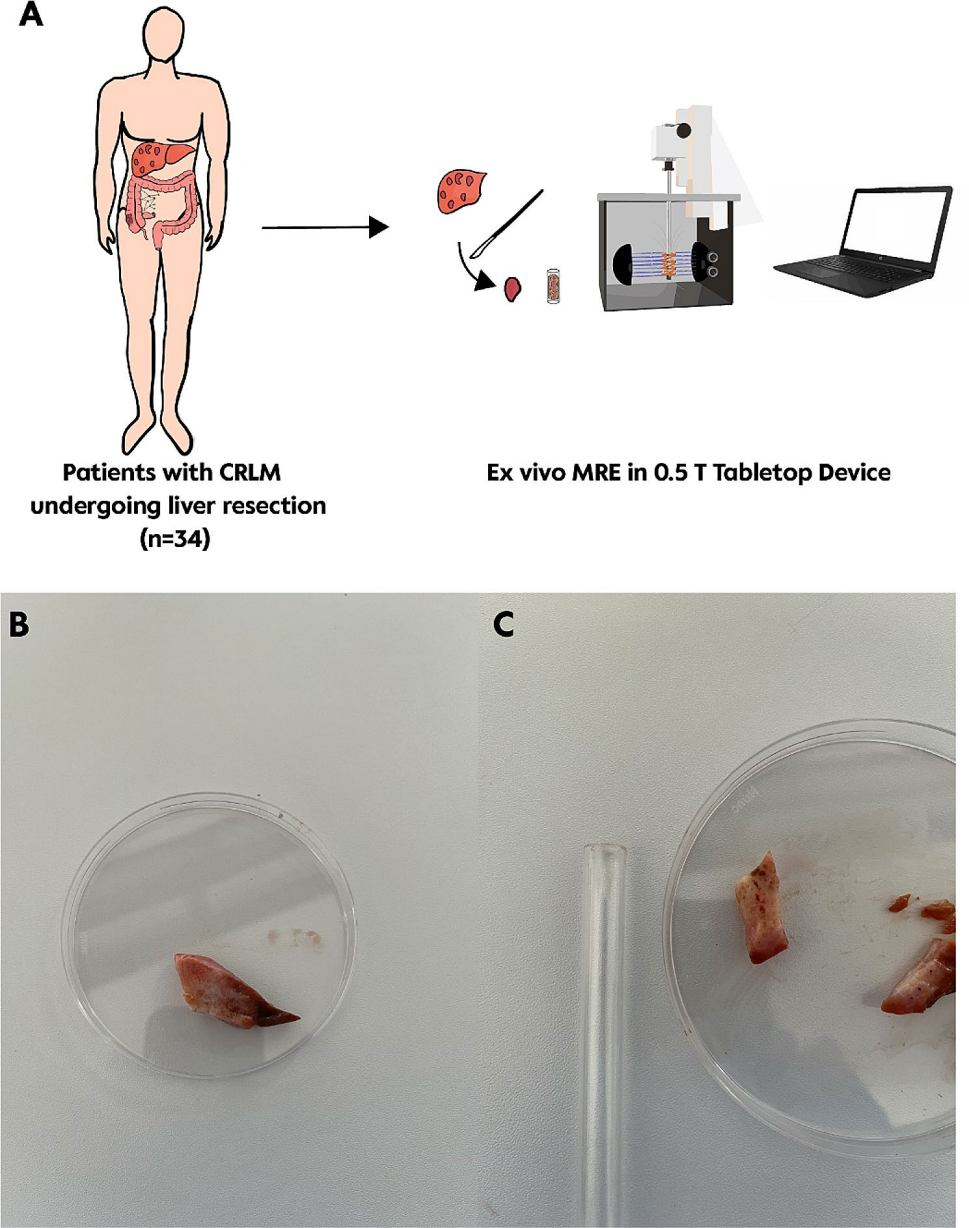
Schematic overview of the study design (**A**). Macroscopic view of a CRLM sample (**B**). Dissection of specimen with 7 mm diameter next to the samples tube for the MRE measurement (**C**)

The CRLM samples were measured within a maximum timeframe of 24 h after sample collection and thoroughly warmed to a temperature of 26 °C before the measurements to minimize possible changes in the viscoelastic tumor properties (R 2.3, 21, 22). The MRE measurements were conducted utilising a custom-built ex vivo tabletop MRE set-up consisting of a compact 0.5 Tesla MRI scanner (Pure Devices GmbH, Würzburg, Germany), a four-channel external gradient amplifier (DC 600, Pure Devices GmbH) and an integrated piezoelectric actuator (PAHL60/20 Piezosystem Jena GmbH, Jena, Germany). A detailed description of the set-up is outlined by *Braun et al.* and shown in Fig. 1 [18]. The frequency range employed to induce concentric shear waves in the tissue specimens covered 500 Hz to 5300 Hz, incremented by 400 Hz intervals. This frequency modulation scheme has been previously utilized for the analysis of liver samples [20]. The shear waves travelled concentrically from the walls of the glass tube towards the centre of the sample with a polarization direction along the main axis of the cylindrical tube. The sample tube had an inner diameter of 7 mm and a height of 200 mm. The tissue samples were cylindrically cut to a diameter of 7 mm to facilitate insertion into the tube. The approximate time between storage at 4 °C and the actual measurement was 15 min, where the samples could adapt to the room temperature.

Wave images were acquired with a spin-echo based MRE sequence with trapezoidal bipolar motion-encoding gradients of 0.4 T/m amplitude, synchronized to the vibration frequency and polarized along the main axis of the sample tube. The total motion encoding gradient (MEG) time was set to 20 ms, divided into 10 ms each before and after the refocusing pulse, resulting in a variable number of MEG cycles across all frequencies to fit within the predefined time interval. Four time offsets were acquired over a vibration period to capture the wave propagation by generating multiple images. T1 relaxation times for each sample were previously determined in a range between 700 and 1200 ms and taken for the MRE sequence as repetition time. Other sequence parameters were echo time 7.5 ms, matrix size 64 × 64, in-plane resolution 150 × 150 µm^2^, slice thickness 3 mm. One central slice image was taken for each CRLM sample. Overall, the acquisition time was approximately 60 min per tissue.

The tissues were investigated at a constant temperature of 26 °C, regulated by a temperature control unit integrated in the compact MRI scanner. Following the measurements, all CRLM samples were portioned into two pieces and stored at -80 °C for the histological evaluation.

### MRE postprocessing

All imaging data were postprocessed with algorithms written in MATLAB (R2019b, The Mathwork Inc., Natick, MA, United States). After phase unwrapping und Fourier transformation in time, complex-valued wave images of each frequency were fitted by an analytical solution of the wave equation. Therefore, the radial profiles were prescribed into a z-infinite cylinder and fitted by Bessel functions with complex wave numbers k*=k’+ik’’. Shear wave speed *c* (in m/s) and shear penetration rate *a* (in m/s) as proxies for stiffness and viscosity for each frequency were derived by.

1 $$\:c=\frac{\omega\:}{{\text{k}}^{{\prime\:}}}\:\:a=-\frac{\omega\:}{2{\uppi\:}\:{\text{k}}^{{\prime\:}{\prime\:}}}$$

Furthermore, the viscoelastic spring-pot model (SP) was applied to derive rheological, frequency-independent parameters, consisting of *µ* (shear modulus, in Pa) and α(dimensionless powerlaw exponent), as described elsewhere [18]. *µ* directly represents stiffness, while quantifies the viscoelastic dispersion slope of stiffness and viscosity over frequency. α can range between 0 and 1 constituting the two limits of either pure elastic-solid or viscous-liquid behavior, respectively.

### Histological analysis

**Fig. 2:**
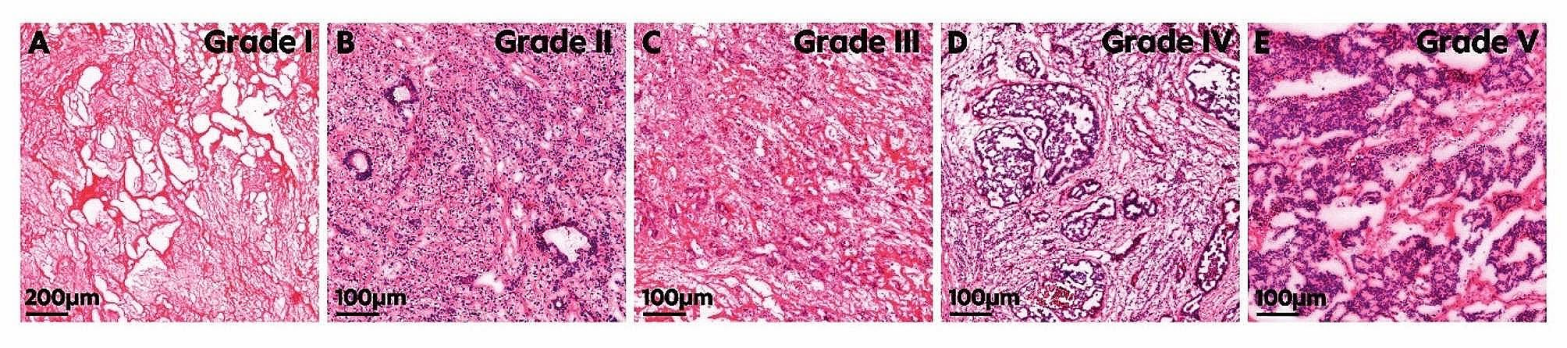
Representative histological slices for each grade of regression. (**A**) and (**B**) major response. (**C**) partial response. (**D**) and (**E**) no response

Cryo-slices with a thickness of 10 µm were prepared from 34 CRLM samples utilizing a Cryostat (CryoStar NX70, Thermo Fisher Scientific Inc., Waltham, MA, United States). The slices were then stained using a Hematoxylin and Eosin staining according to manufactures instruction. The slices were then analysed by a board-certified pathologist regarding their regression grade according to the Rubbia-Brandt classification [23]. Representative histological slices for each tumor regression grade are shown in Fig. 2. These are also the slices we used for the further histological analysis. The histopathological response was defined as followed: Rubbia-Brandt scores “one” and “two” were defined as histopathological major response, a Rubbia-Brandt score of “three” corresponded with a histopathological partial response and Rubbia-Brandt scores “four” and ”five” were defined as histopathological no response. Additionally, the CRLM samples were categorized based on their cell viability and stratified into 11 groups with intervals of 5% or 10% viability.

For the quantitative histology, automatic analysis using image segmentation algorithms written in Python (Version 3.10, Phyton Software Foundation, Wilmington, Delaware, United States) were used. Due to computational optimisation the high-resolution slices were cut into up to about fifty 5000 × 5000 pixels (1105 μm × 1105 μm) tiles by using the OpenSlide library.

The segmentation of the single nuclei was done with the use of a pre-trained model for H&E staining from the StarDist algorithm, which detected star-convex shapes [24, 25]. A probability threshold of 0.8 was used to aid the detection of clustered cell nuclei. Although, only about 10–30% of the nuclei could be automatically detected, we considered their area and aspect ratio as representative for the whole slice. If an automatic detection was not possible, it was manually performed on a random basis. The area (in µm^2^) of each nucleus was calculated by using the regionprops functionality from the skimage.measure package. The aspect ratios of the nuclei were determined by dividing the length of the major axis by the length of the minor axis [26].

Nucleus density (in 1/µm^2^) was derived from the ratio of total nucleus area from clustered and single nuclei to tissue area, which was divided by the average area of the single nuclei segmented by StartDist. We were able to include and analyze 100% of the available nuclei. Therefore, the nucleus area was segmented by first extracting the hematoxylin colour channel from the skimage.color package and performing a gamma correction by using the skimage.exposure package afterwards. The images were then converted into greyscale and were binarized by using a threshold based on Otsu’s method from the skimage.filters package. Connected regions were then labelled with the use of the skimage.measure package.

### Radiological evaluation

The radiological response of the CRLM was assessed before and after chemotherapy using the images from a contrast-enhanced CT scan or contrast-enhanced MRI. A maximum of two pathological liver lesions per patient were analysed. Tumour response was evaluated using the same imaging modalities and RECIST criteria version 1.1, as outlined before [27]. Patients exhibiting a reduction of at least 30% in the diameter of the target lesion were classified as *“radiological partial response”* (rPR), while those displaying an increase of at least 20% in the target lesion diameter were categorized as *“radiological progressive disease”* (rPD). All other were grouped as *“radiological stable disease”* (rSD).

### Statistical analysis

Statistical analysis was performed using *R* (version 4.3.0) and R Studio (version 2023.06.0) for macOS (both R Foundation for Statistical Computing, Vienna, Austria). We used the packages tidyverse, gtsummary, data.table, Hmisc, tidyr, dplyr, and cutpointr for the analyses and visualization. For the normality testing we used the Shapiro-Wilk-Test. Continuous variables were compared using ANOVA or Kruskal-Wallis Test. Non-parametric variables are reported as median and interquartile range (IQR). Correlation analyses were performed by using the Pearson correlation coefficient or Spearman’s rank correlation coefficient depending on the variable and results of the normality testing. A p-value ≤ 0.05 was considered significant.

## Results

### Baseline characteristics of the cohort

34 CRLM samples from 31 patients with a median age of 65 years (range: 32–82 years) were analysed in this study (Table 1). We were able to obtain two samples from different lobes of the same livers at different time points from three patients who were undergoing staged hepatectomy. Predominantly, the patients had left-sided colorectal cancer as a primary cancer localisation (76.5%) and only 17.6% right-sided colon cancer. One patient was initially diagnosed with a cancer of unknown primary origin due to no diagnostic findings in the primary colonoscopy, which was later described to be CRC in the histopathological report. Another patient’s history did not further specify the exact localisation of the primary CRC site. Based on the initial TNM-Classification, most of the patients had a far advanced CRC, including invasion of nearby structures, regional lymph node invasion and synchronous metastases. The latest administrated chemotherapy regimen prior to the resection of the CRLM is presented in Table 1. Only 6 of 34 (17.6%) CRLM samples did not receive preoperative chemotherapy before resection. The most common regime for chemotherapy contained irinotecan, including FOLFIRI and FOLFOXIRI (50%), following the use of oxaliplatin (20.6%). Regimens that did not include either of these agents included, for example, the exclusive administration of 5-FU. 61.8% of the patient samples received an additional targeted therapy with either Anti-VEGF (26.5%) or Anti-EGFR antibodies (35.3%). Most patients underwent major hepatic resection involving three or more liver segments (70.6%) with a minimal invasive approach in 73.5% of the surgical procedures.

**Table 1:** Preoperative characteristics of 34 samples from patients with CRLM that underwent partial liver resection

Characteristics	Samples from patients with CRLM (*n* = 34)
Sex, n (%)	
Female	13 (38.2)
Male	21 (61.8)
Median age at resection in years, (range)	64.5 (32–82)
Localization of primary cancer, n (%)	
Right-sided colon cancer	6 (17.6)
Left-sided colorectal cancer	26 (76.5)
No localization documented	2 (5.9)
Initial TNM-Classification, n (%)	
Locally restricted primary tumour (T < 3)	3 (8.8)
Invasion of nearby structures by primary tumour (T ≥ 3)	29 (85.3)
Regional lymph node invasion	21 (61.7)
Synchronous metastases	21 (61.9)
Initial classification not specified	2 (5.9)
Last chemotherapy regime before CRLM resection, n (%)	
No preoperative chemotherapy	6 (17.6)
Regime with Oxaliplatin	4 (11.8)
Regime with Irinotecan	14 (41.2)
Regime with combination of both	3 (8.8)
Regime with none of the above	6 (17.6)
Regime not specified	1 (2.9)
More than one chemotherapy regime	13 (38.2)
Additional targeted therapy	
No targeted therapy	13 (38.2)
Anti-VEGF antibodies	9 (26.5)
Anti-EGFR antibodies	12 (35.3)
Surgical approach, n (%)	
open	9 (26.5)
minimal invasive surgery	25 (73.5)
Surgical procedure, n (%)	
Major	24 (70.6)
Minor	10 (29.4)

### Histopathological analysis

All samples were grouped according to their regression grades and the amount of vital tumour cells in the tissue were quantified (Table 2**)**. Three samples were grouped into grade 1 of Rubbia-Brandt and two samples into the grade 2 of the tumour regression score, as a result showing major response to chemotherapy (14.7%). Six samples showed partial response, transcribing into a grade 3 of Rubbia-Brandt (17.6%). Fifteen of the samples were grouped into grade 4 of the tumour regression score and eight samples into grade 5, therefore showing no response (67.6%). We were able to get a deeper understanding of the distribution regarding the proportion of the vital tumour cells within the regression scores. It was noticeable that these groups overlapped to some extent with the regression grades, probably due to the manual categorization. Tumour samples with major response to chemotherapy exhibited ≤ 10% proportion of vital tumour cells, whereas samples of partial response contained vital tumour cells between 10 and 50%. Tissue samples characterized as non-responders harboured at least 60% of vital tumour cells, including CRLM without preoperative chemotherapy. Specifically, four of these samples exhibited 80% vital tumour cells, while two samples contained 100%.

**Table 2:** Summary of the analysis of the regression grade, with explanation for each grade and the corresponding vital tumour cell subgroups

Tumour regression grade by Rubbia-Brandt	Number of samples for each grade (*n* = 34)	Explanation of each regression grade	Amount of vital tumour cells (*n*)
1	3	Absence of residual tumour cells and large amount of fibrosis	0% (2) 5% (1)
2	2	Rare residual cancer cells scattered throughout the fibrosis	5% (1) 10% (1)
3	6	More residual cancer cells but with predominating fibrosis	10% (1) 20% (2) 30% (1) 50% (2)
4	15	Residual tumour cells predominate over fibrosis	60% (1) 70% (3) 80% (10) 90% (1)
5	8	No signs of regression	80% (1) 90% (4) 100% (3)

**Fig. 3:**
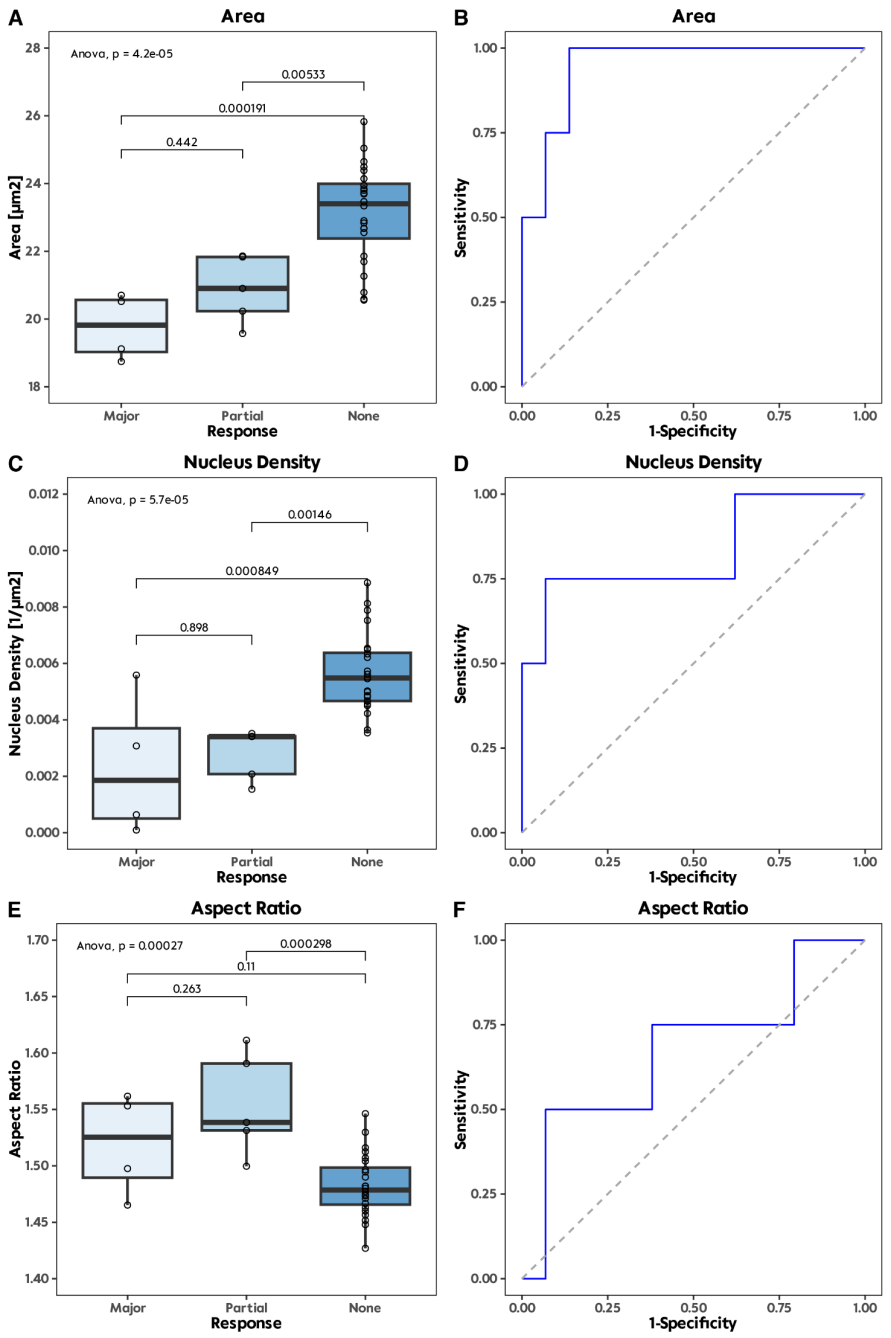
Boxplots of the quantitative histology for cell area (in µm2) (**A**), nucleus density (in 1/µm2) (**C**) and aspect ratio (**E**) regarding the response to chemotherapy. The corresponding calculations of the AUC for the quantitative parameters are depicted in the second row (**B**, **D**, **F**).

Results of quantitative histology are summarized in Fig. 3. We observed significant differences in nucleus area (*p* < 0.0001), particularly when comparing major response to no response (19.82 µm^2^, IQR 18.84, 20.66 vs. 23.4 µm^2^, IQR 22.03, 24.09, *p* = 0.0002), as well as between no response and partial response (23.4 µm^2^, IQR 22.03, 24.09 vs. 20.9 µm^2^, IQR 19.9, 21.85, *p* = 0.0053).

In the evaluation of the nucleus aspect ratio, we could only observe a significance comparing samples with no and partial response (1.49, IQR 1.46, 1.50 vs. 1.54, IQR 1.52, 1.61, *p* = 0.0003).

By analysing the nucleus density in response to chemotherapy among CRLM samples, we identified notable differences (*p* < 0.0001). Specifically, we observed significant differences between subsets exhibiting major response (0.0019 /µm^2^, IQR 0.0002, 0.0050) compared to those with no response (0.0055 /µm^2^, IQR 0.0047, 0.0065, *p* = 0.0008). Furthermore, a distinct contrast was evident between partial response (0.0034 /µm^2^, IQR 0.0018, 0.0035) and no response (0.0055 /µm^2^, IQR 0.0047, 0.0065, *p* = 0.0015).

Calculation of AUC for major vs. non-major response depicted for nucleus area 0.95 (sensitivity: 1 and specificity: 0.86), for aspect ratio 0.67 (sensitivity: 0.5 and specificity: 0.93) and for nucleus density 0.83 (sensitivity: 0.75 and specificity: 0.93).

### Radiological evaluation

We were not able to evaluate the response from seven patient samples due to clinically not available data. This included no preoperative chemotherapy and missing imaging data after chemotherapy, respectively. A total of 10 patients were grouped into rPR, 14 into rSD and 3 into rPD. The results derived from the evaluation of radiological and pathological response are compared in Table 3.

**Table 3:** Assessment of radiological and pathological response in 27 samples from patients with CRLM, n (%)

	Pathological response	
	**Absence** (no or partial response)	**Presence** (major response)
Radiological response		
Absence (stable or progressive disease)	16 (59.2)	1 (3.7)
Presence (partial response)	6 (22.2)	4 (14.8)

The majority of the patients (20/27, 74%) showed a concordance in pathological and histological response. Four patients (14.8%) showed a presence in both responses and 16 patients (59.2%) showed no response in either assessment. Interestingly, a total of seven patients (25.9%) showed a discordant radiological and pathological response evaluation. One patient (3.7%) showed a response in the pathological assessment but no response in the radiological evaluation while six patients (22.2%) showed a presence in the radiological response and an absence in the pathological response.

### MRE measurements

**Fig. 4:**
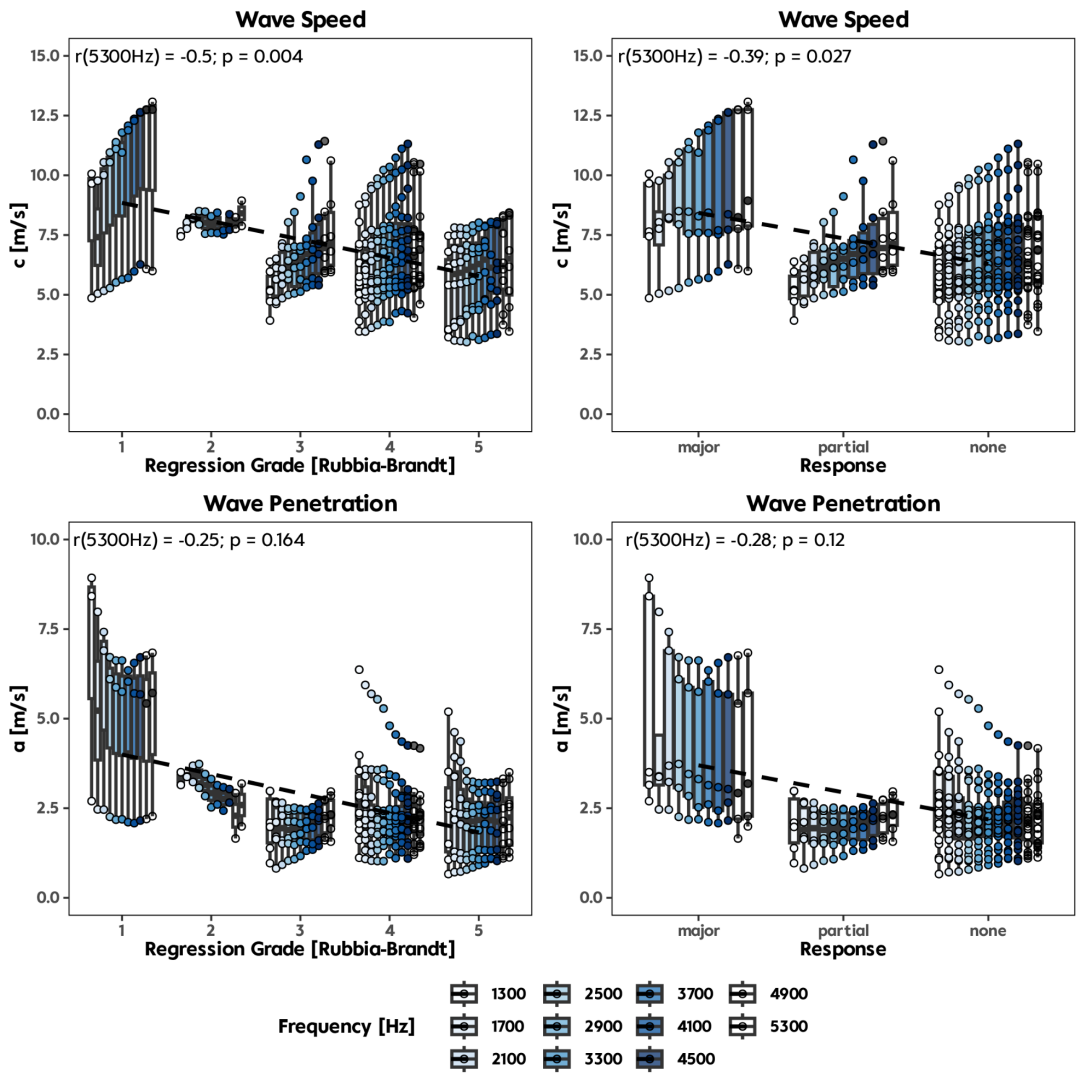
Correlation analysis for shear wave speed (**c**) and tumour regression score (top left), shear wave speed and histological response to chemotherapy (top right), wave penetration (**a**) and tumour regression score (lower left) and wave penetration and histological response (lower right).

34 out of 46 studied CRLM samples could be evaluated successfully. 12 tissue specimens (35.3%) had to be measured twice and one sample (2.9%) three times due to technical difficulties, including insufficient wall contact of the sample. The median time for the start of the measurements after obtaining the samples was 100 min (range between 24 min and 19 h). 15 samples (42.9%) were investigated within one hour, 12 samples (35.3%) within two and the rest (20.6%) within more extended periods of at maximum 19 h. Shear wave speed *c* and penetration rate *a* were calculated for all measured samples for the vibration frequencies 500–5300 Hz. However, the first two frequencies (500 Hz and 900 Hz) had to be excluded due to amplitude overshot or too long wavelengths. Wave lengths exceeding the sample diameter by a factor of two were considered as a limit for tabletop MRE [18]. We grouped the tissue samples based on their regression grade and their histopathological response to chemotherapy. We were able to show a significant correlation for *c* with the regression grade for the frequencies including 1300, 2100 Hz and above (Fig. 4 and Table 4**)**. Shear wave speed values were higher at higher regression grades according to Rubbia-Brandt scores one and two indicating low cell viability. No correlation between penetration rate *a* and regression grade was observed (*p* > 0.05 for all frequencies). When the samples were classified according to their histopathological response, we observed a significant correlation for *c* at 4900 Hz and 5300 Hz (*p* = 0.030 and *p* = 0.027, respectively). These results indicate that CRLM samples with a major response to chemotherapy have a higher stiffness than samples with partial or no response to chemotherapy.

**Table 4:** Summary of correlation analysis for *a* and *c* regarding the tumour regression grade and response to chemotherapy for each frequency

**Classifier**	**Frequency**	Wave Penetration (a)		Wave Speed (c)		
		**Correlation Coefficient**	**p**	**Correlation Coefficient**	**p**	
Regression Grade	1300	-0.241	0.183	**-0.441**	**0.011**	
Regression Grade	1700	-0.110	0.541	-0.283	0.110	
Regression Grade	2100	-0.248	0.157	**-0.435**	**0.010**	
Regression Grade	2500	-0.265	0.130	**-0.449**	**0.008**	
Regression Grade	2900	-0.260	0.138	**-0.441**	**0.009**	
Regression Grade	3300	-0.251	0.153	**-0.443**	**0.009**	
Regression Grade	3700	-0.255	0.146	**-0.447**	**0.008**	
Regression Grade	4100	-0.227	0.197	**-0.438**	**0.010**	
Regression Grade	4500	-0.245	0.162	**-0.465**	**0.006**	
Regression Grade	4900	-0.252	0.150	**-0.475**	**0.005**	
Regression Grade	5300	-0.252	0.164	**-0.500**	**0.004**	
Response	1300	0.198	0.278	0.242	0.181	
Response	1700	0.098	0.587	0.146	0.417	
Response	2100	0.277	0.113	0.303	0.081	
Response	2500	0.287	0.100	0.317	0.068	
Response	2900	0.280	0.109	0.266	0.128	
Response	3300	0.249	0.156	0.298	0.087	
Response	3700	0.259	0.139	0.306	0.079	
Response	4100	0.223	0.204	0.275	0.115	
Response	4500	0.233	0.184	0.334	0.054	
Response	4900	0.235	0.181	**0.373**	**0.030**	
Response	5300	0.283	0.116	**0.392**	**0.027**	

**Fig. 5:**
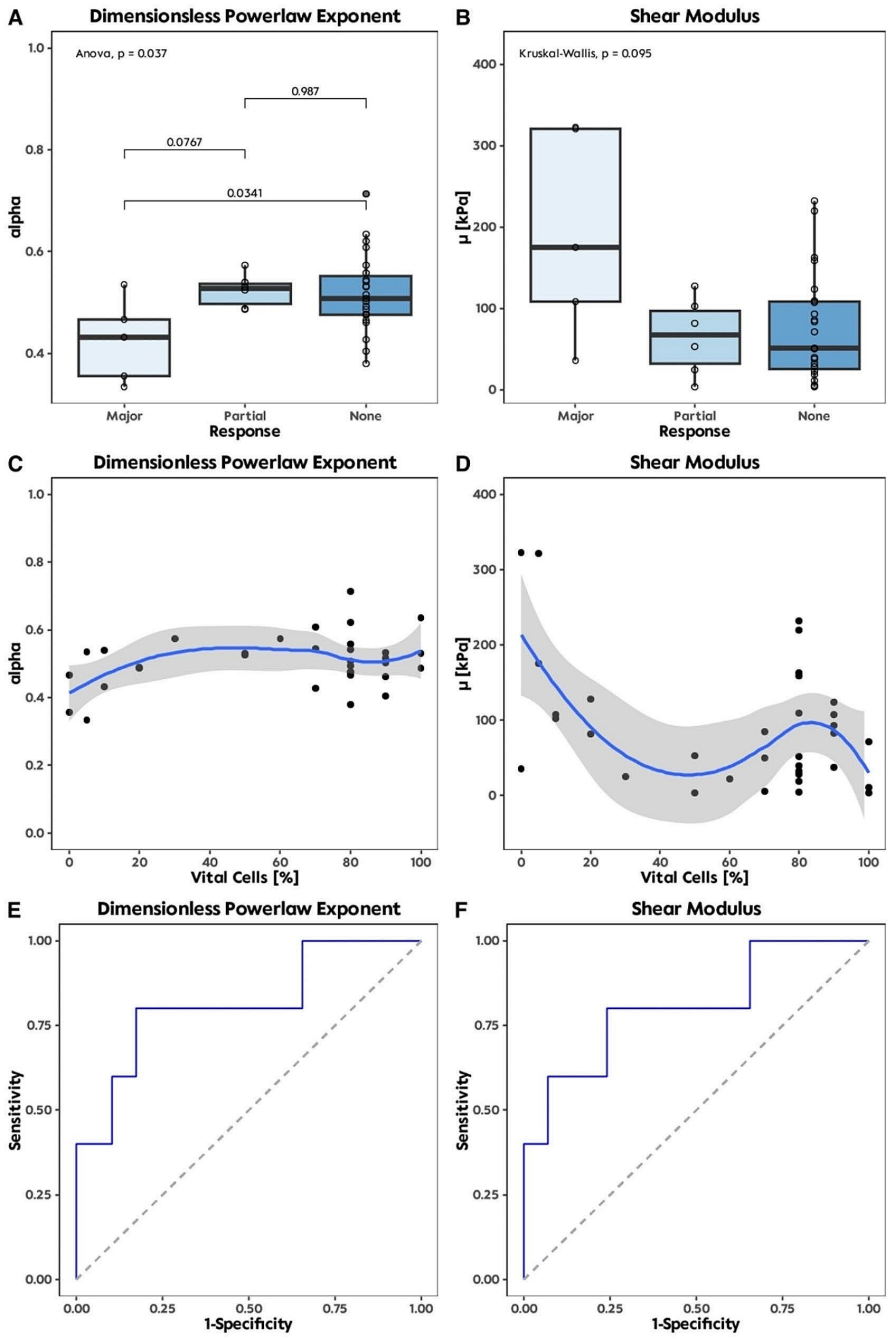
Boxplots of the frequency-independent parameters of the SP-model for the histological response to chemotherapy (**A**, **B**). Distribution of µ and ɑ dependent on the amount of vital tumour cells within the tissue (C, D). Calculation of diagnostic accuracy for the SP parameters on the basis of AUC (**E**, **F**).

Frequency-independent parameters *µ* and *α* of the SP-model are shown in Fig. 5. *α* was significantly lower in samples with a major response (0.41, IQR 0.36, 0.47) compared to samples with no response (0.51, IQR 0.48, 0.55; *p* = 0.03) (Fig. 5A). Samples with histopathological partial or no response had a median *α* above 0.5 (0.53, IQR 0.50, 0.54 vs. 0.51, IQR 0.48, 0.55), depicting a more viscous-fluid behaviour. Additionally, samples with histopathological major response showed a median *α* < 0.5 (0.43, IQR 0.36, 0.47) indicating more elastic-solid tissue properties, while *µ* was not significantly altered (*p* = 0.095). The diagnostic accuracy for the prediction of the major response according to the *α* and *µ* values is shown in Fig. 5E, F with AUC of 0.82 for *α* (sensitivity: 0.86 and specificity 0.8) and 0.83 for *µ* (sensitivity: 0.8 and specificity: 0.93). However, since we saw no significant differences for *µ*, the AUC should be considered more as a trend for the diagnostic accuracy.

## Discussion

With the advent of multimodal therapeutic approaches for patients diagnosed with colorectal liver metastases, significant enhancements in patient outcomes have been achieved. Nonetheless, opportunities for further improvement persist, underscoring the need for more personalized treatment strategies. For this reason, optimized imaging modalities are required to better evaluate and monitor the response to chemotherapy.

To the best of our knowledge, this is the first study analysing the viscoelastic properties of CRLM with ex vivo MRE in tissue specimens and investigating the relationship between response to chemotherapy, tumour cell viability and the related changes in biomechanical tissue properties. Our results indicate that ex vivo MRE might be able to detect the changes in the biomechanical properties associated with cell viability and regression grade of CRLM. We were able to show that tissue samples from CRLM have an overall higher stiffness compared to healthy liver tissue [21] and we also confirmed the in vivo results from *Vogl et al.* by showing that stiffness of CRLM increases with systemic chemotherapy, which correlates with major response [16]. In patients with advanced hepatocellular carcinoma (HCC) treated with pembrolizumab as immunotherapy, an increased stiffness was associated with a better overall survival and prolonged time to progression [28]. These results are in line with our findings and support the potential use of in vivo MRE to continuously monitor the response to chemo- or immunotherapeutic treatment.

Many aspects influence the tissue stiffness itself and the composition of the viscoelastic properties [16]. Our observation of pronounced viscous-fluid properties at higher Rubbia-Brandt scores, which indicate partial or no response to chemotherapy, may be explained by the fact, that a high regression score results from high tumour cell viability and low amount of fibrosis [9]. The higher *α*-values in our group of non-responders might indicate the ability of tumour cells to act collectively and to organize into multicellular streams, which increases the fluidity of the tissue on a coarse-grained scale [29]. A similar behaviour has been observed in metastatic cancer cells, where cell unjamming enhanced the fluidity of tissues and enabled them to migrate into other areas [29, 30]. Similarly, cells within CRLM samples showing no response to chemotherapy might possess the capability to proliferate and migrate within and beyond their tumour clusters. Conversely, the higher amount of fibrosis as well as dead tumour cells in response to chemotherapy might contribute to tissue stiffening and reduced viscous loss and tissue fluidity. Our findings showed that CRLM responders exhibit high stiffness and elastic-solid behaviour, which is consistent with prior research demonstrating that fibrotic liver tissue has similar viscoelastic properties [31]. This suggests a biomechanical and cytological similarity between theses tissues. Moreover, the microenvironment of the tumour plays a major role in influencing the capability of tumour growth and invasion by regulating the pathways for epithelial to mesenchymal transition [32]. A stiffer extracellular matrix (ECM) is associated with an increased ability for tumour cells to migrate [32]. In addition to that, matrix stiffness can also alter the sensitivity to chemotherapy. *Shen et al.* observed a higher stiffness in CRLM compared to the primary colorectal tumour and further metastatic stiffening caused a reduction in the sensitivity to treatment [33]. It would be interesting to investigate whether the microenvironment of the tumour itself or the cellular composition of the tissue has a more substantial influence on stiffness. Our quantitative histologic analysis showed that CRLM samples with major response displayed an increase of stiffness with decreasing nucleus area and nucleus density suggesting that ECM alterations and the structure of cell clusters both influence the mechanical response in our samples.

In contrast to the findings reported by *Brouquet et al.* (44%) we did not observe a similarly high level of discordance between pathological and radiological assessments in our samples (26%). This difference could be attributed to our smaller sample size and the preselection process, as not all resected CRLM specimens were suitable for the MRE analysis [9]. Most of our samples showed neither a pathological nor a radiological response, while only a subset depicted both a radiological and histological response. The question arises as to whether MRE could potentially minimize this discordance by providing additional valuable information to complement the radiological results.

In the CAMINO-Trial, the surgical therapy plan was altered in 31% of the patients after contrast-enhanced MRI, in several cases to a less extensive therapy regimen [8]. Therefore, gaining a comprehensive understanding of the biological characteristics of CRLM using MRI may be crucial, as a discordance between pathological and radiological response is associated with a reduced disease-free survival. MRE in combination with standard MRI might address this problem by allowing us to monitor patients with CRLM throughout the course of chemotherapy by identifying those patients with radiological response but pathological non-response, one could tailor the preoperative systemic chemotherapy approach more specifically on an individual base.

*Li et al.* demonstrated that an early radiological evaluation of treatment response may also have a predictive value [34]. Their group investigated the use of spectral computed tomography (CT) to evaluate the treatment response in patients suffering from CRLM and being treated with bevacizumab in combination with FOLFOX or FOLFIRI two months after the start of first-line treatment. With this approach, they were able to predict the overall survival and response. However, due to the anti-angiogenic effect of bevacizumab, vascularization of CRLM is suppressed and contrast enhancement is reduced. Therefore, the strong predictive effect of the spectral CT might be limited to bevacizumab-containing chemotherapy regimens.

It is important to acknowledge that adenocarcinomas, particularly CRC, comprise a very heterogeneous group of histological variants [35]. Therefore, comparing different variants with each other could pose challenges, as it is conceivable that, for instance, a mucinous and an undifferentiated CRC, may have different viscoelastic tissue properties. To avoid this problem, it would have been ideal to measure the same tumour prior to the chemotherapy as well as at different cycles. We were able to analyse two samples from each of three patients at different time points. However, they all had the same tumour regression grade after the first and second surgical procedure. Furthermore, we believe that we would have had more heterogeneous results within the subgroups if the viscoelastic properties differed significantly between the histologic variants. In patients with HCC, the stiffness of well and moderately differentiated tumours differed significantly from patients with undifferentiated tumours [36]. It could be hypothesized that the tumour phenotype or the histological growth pattern of CRLM also affects the viscoelastic properties. However, this needs to be further investigated.

Although we have obtained interesting and promising results, our study has some limitations. Due to the study design, we could only determine the grade of regression after the MRE measurements, resulting in very heterogenous group sizes. One reason for the smaller group sizes, especially in the groups with major response to chemotherapy, could be related to the limited amount of resected tissue. The clinical protocol prioritized pathological examination, which sometimes resulted in inadequate tissue samples for MRE measurement. These tissue samples could have shown major response to chemotherapy since a decrease in the diameter of a tumour is associated with radiological response and therefore to chemotherapy [27].

Furthermore, the CRLM samples were measured mostly within 2 h after resection. However, a subset of the samples (20.6%) underwent measurement after an extended period, ranging between 3 and 19 h, though all measurements occurred within 24 h following sample collection. Ideally, standardizing the measurement timepoint for all samples would have enhanced consistency and comparability across the dataset. A study by *Garczynska et al.* showed that the stiffness of liver tissue increases within a timeframe of 20 min due to blood coagulation. While after this initial phase and up to 17 h, the stiffness of the samples remained relatively stable at a temperature of 26 °C [21]. Therefore, the specimens in our study were stored at 4 °C and measured within 24 h to minimize changes in the tissue structure. Furthermore, in order to optimize the position of the specimens in the glass tube, one third of the samples were examined two or three times by MRE within 2–3 h after resection. Encouragingly, we did not saw any change in the viscoelastic properties of these samples.

For the future, we plan to pair our ex vivo MRE setup with in vivo MRE measurements prior to chemotherapy and surgery, to see if the results are comparable to the lower frequency range of in-vivo MRE and if the cell viability can be assessed in patients. Once this setup is translated into the clinic, MRE could be used to improve the diagnostic and therapeutic options for patients with CRLM. The combination of standard preoperative MRI and MRE could potentially help to better predict the response of CRLM to systemic chemotherapy than MRI alone. Ultimately, this could provide an option to non-invasively access the regression grade of the tumour before liver resection and initiate changes to the systemic chemotherapy towards individually tailored therapies.

## Conclusion

In summary, ex vivo MRE was used to characterize the viscoelastic properties of CRLM samples and to stratify their responses to chemotherapy. We were able to show that CRLM with histological major response to chemotherapy have a significant higher shear wave speed as a marker of tissue stiffness and low cell viability. In addition, CRLM responders showed a pronounced elastic-solid behaviour compared to the ones with partial or no response. Histological analysis confirmed that the viscoelastic tissue properties were influenced by cell viability and ECM components, especially the extent of fibrosis. In the future both in vivo and ex vivo MRE prior and during chemotherapy could be used to tailor therapeutic approaches based on the biomechanical response of CRLM to treatment.

## Data Availability

The datasets during and/or analyzed during the current study available from the corresponding author on reasonable request.

## Abbreviations

CRC: Colorectal cancer
CRLM: Colorectal liver metastases
CT: Computed tomography
ECM: Extracellular matrix
HCC: Hepatocellular cancer
MEG: Motion encoding gradient
MRE: Magnetic resonance elastography
MRI: Magnetic resonance imaging
rPD: Radiological progressive disease
rPR: Radiological partial response
rSD: Radiological stable disease
SP: Spring-pot model
